# Responses to the comment: Anxiety and depression among Chinese adolescents during the COVID-19: an overestimation of the problem

**DOI:** 10.1038/s41398-022-01826-z

**Published:** 2022-02-17

**Authors:** Rui Liu, Han Qi, Xu Chen, Teris Cheung, Todd Jackson, Gang Wang, Yu-Tao Xiang

**Affiliations:** 1 The National Clinical Research Center for Mental Disorders & Beijing Key Laboratory of Mental Disorders, Beijing Anding Hospital & the Advanced Innovation Center for Human Brain Protection, Capital Medical University, Beijing, China; 2 School of Nursing, Hong Kong Polytechnic University, Hong Kong SAR, China; 3 Department of Psychology, University of Macau, Macao SAR, China; 4 Unit of Psychiatry, Department of Public Health and Medicinal Administration, & Institute of Translational Medicine, Faculty of Health Sciences, University of Macau, Macao SAR, China; 5 Center for Cognition and Brain Sciences, University of Macau, Macao SAR, China; 6 Institute of Advanced Studies in Humanities and Social Sciences, University of Macau, Macao SAR, China

**Keywords:** Depression, Scientific community

**Dear Editor,**

We thank Kojak et al. for their comments [1] on our recently published paper “Depression, anxiety and associated factors among Chinese adolescents during the COVID-19 outbreak: a comparison of two cross-sectional studies” [2]. Below are our responses to their remarks.

In this study featuring two separate surveys, public education on mental health and psychosocial interventions was offered to many of the adolescents who reported depression and anxiety by local health authorities in some areas following the first survey; therefore, those who completed both surveys were removed from analyses because retaining them could bias results of the second survey as noted by one reviewer of the initial submission. Notably, however, even if those who completed both surveys are included for analyses, the results are quite similar to the primary findings reported in our paper.

Compared to prevalence rates of depression and anxiety from the first survey, we found that the corresponding figures in the second survey increased (or were higher). The terms, “increased” and “higher”, can be used interchangeably here as both refer to upward trends. The two terms have been widely used in similar epidemiological surveys.

If Kojak and colleagues had read the paper carefully, they would have found that we did not describe depression and anxiety as “disorders”. Indeed, in the limitations section, we had explicitly stated that standardized diagnostic instruments such as the Structured Clinical Interview for the DSM-IV (SCID) could not be used due to safety requirements associated with minimizing face-to-face contacts during the COVID-19 pandemic. Furthermore, depression and anxiety are general terms in clinical practice and research that are used conventionally to describe disorders as well as symptoms.

The cutoff values of the Center for Epidemiological Studies-Depression Scale (CES-D) and the 7-item Generalized Anxiety Disorder (GAD-7) scale used in this study were based on validation studies of these scales conducted in China that we cited in the paper. Due to different socio-cultural contexts, cutoff values suggested in original validation studies of the scales are less appropriate for use in China. Kojak and colleagues mentioned GAD-7 cutoff values of 6 and 7 were used in samples of Chinese people with epilepsy and Chinese pregnant women. Cut-offs based on samples of non-adolescents are not sufficient as cut-offs for adolescents. Additionally, it is general knowledge that pooled scale cutoff values in meta-analyses and systematic reviews based largely on samples drawn from Western countries should not be applied globally to particular non-Western populations due to differing socio-cultural contexts between countries.

We certainly agree that unmeasured factors were potentially associated with depression and anxiety in our sample. Once again, we noted this clearly with associated reasons in the limitations section of our paper. In this study, sleep duration, study duration, exercise duration, study efficiency, and concerns about entering a higher grade were included as items in the assessment of socio-demographic background and clinical characteristics of respondents. If Kojak and colleagues had attended to the “Method” section carefully, they would have found this was introduced.

Finally, this was an exploratory study, rather than a confirmatory study designed to test a specific theory or model; therefore, a priori hypotheses were not required or generated. Furthermore, as noted in the “Method” section of the paper, data were collected using the smartphone-based WeChat–Wenjuanxing application. Participants who chose to not complete all survey items could not successfully submit their responses to the researchers.
